# Effect of Dietary Docosahexaenoic Acid Supplementation on the Participation of Vasodilator Factors in Aorta from Orchidectomized Rats

**DOI:** 10.1371/journal.pone.0142039

**Published:** 2015-11-05

**Authors:** Diva M. Villalpando, Rocío Navarro, Lara del Campo, Carlota Largo, David Muñoz, María Tabernero, Ramiro Baeza, Cristina Otero, Hugo S. García, Mercedes Ferrer

**Affiliations:** 1 Departamento de Fisiología, Facultad de Medicina, Universidad Autónoma de Madrid, Madrid, Spain; 2 Área Cardiovascular, Instituto de Investigación Hospital Universitario La Paz (IdiPAZ), Madrid, Spain; 3 Cirugía Experimental, Instituto de Investigación Hospital Universitario La Paz (IdiPAZ), Madrid, Spain; 4 Gabinete Veterinario, Facultad de Medicina, Universidad Autónoma de Madrid, Madrid, Spain; 5 Instituto de Catálisis y Petroleoquímica, Consejo Superior de Investigaciones Científicas, Madrid, Spain; 6 Instituto Tecnológico de Veracruz, Veracruz, México; Max Delbrueck Center for Molecular Medicine, GERMANY

## Abstract

Benefits of n-3 polyunsaturated fatty acids (PUFAs) against cardiovascular diseases have been reported. Vascular tone regulation is largely mediated by endothelial factors whose release is modulated by sex hormones. Since the incidence of cardiovascular pathologies has been correlated with decreased levels of sex hormones, the aim of this study was to analyze whether a diet supplemented with the specific PUFA docosahexaenoic acid (DHA) could prevent vascular changes induced by an impaired gonadal function. For this purpose, control and orchidectomized rats were fed with a standard diet supplemented with 5% (w/w) sunflower oil or with 3% (w/w) sunflower oil plus 2% (w/w) DHA. The lipid profile, the blood pressure, the production of prostanoids and nitric oxide (NO), and the redox status of biological samples from control and orchidectomized rats, fed control or DHA-supplemented diet, were analyzed. The vasodilator response and the contribution of NO, prostanoids and hyperpolarizing mechanisms were also studied. The results showed that orchidectomy negatively affected the lipid profile, increased the production of prostanoids and reactive oxygen species (ROS), and decreased NO production and the antioxidant capacity, as well as the participation of hyperpolarizing mechanisms in the vasodilator responses. The DHA-supplemented diet of the orchidectomized rats decreased the release of prostanoids and ROS, while increasing NO production and the antioxidant capacity, and it also improved the lipid profile. Additionally, it restored the participation of hyperpolarizing mechanisms by activating potassium. Since the modifications induced by the DHA-supplemented diet were observed in the orchidectomized, but not in the healthy group, DHA seems to exert cardioprotective effects in physiopathological situations in which vascular dysfunction exists.

## Introduction

The endothelium plays a pivotal role in the regulation of vascular tone through the release of vasoactive factors such as nitric oxide (NO), prostanoids, reactive oxygen species (ROS) and hyperpolarizing factors [1]. It is well known that NO-induced vasodilation is mediated through the activation of soluble guanylate cyclase, by increasing levels of cyclic guanosin monophosphate (cGMP) and cGMP-dependent protein kinase activity in the vascular smooth muscle of the arterial wall [2,3]. However, vascular functionality of endothelial NO depends on its bioavailability as NO can be metabolized into different ROS.

Prostanoids are synthesized from arachidonic acid metabolism through the cyclooxygenase (COX) pathway. Apart from their involvement in platelet aggregation and inflammation, they are also important regulators of vascular tone in health and disease [1,4]. Hyperpolarizing factors and mechanisms also participate in the regulation of vessel tone by activating potassium channels, which are responsible for the control of membrane potential [2,5]. Calcium-dependent (K_Ca_) and ATP-dependent (K_ATP_) potassium channels are widely expressed in vascular tissue [6,7]. The release of an endothelium derived hyperpolarizing factor (EDHF) has been proposed and while the nature of EDHF remains to be defined, studies have suggested that hyperpolarization may result from endothelial release of different substances such as NO [8], superoxide anion [9] and other ROS [10].

Altered production and/or vascular effects of those factors could induce vascular dysfunction, leading to the development of different cardiovascular disorders such as atherosclerosis, hypertension and ischemia [1,11]. Over the years, different pharmacological treatments have been developed to mitigate the effects of these alterations such as antihypertensives, β-blockers, statins and aspirin [12–14]. Recently, studies have focused on nutritional interventions, with particular interest being paid to n-3 polyunsaturated fatty acids (PUFAs). For example, eicosapentaenoic acid (20:5n-3) or docosahexaenoic acid (DHA; 22:6n-3), that are found in seafood and fish oil supplements, due to their beneficial effects on cardiovascular diseases [15]. Antithrombotic, anti-inflammatory, and vasoprotector effects of PUFAs on the cardiovascular system have been reported [16–18]. Additionally, hyperpolarizing effects of PUFAs, contributing to the vasorelaxation through the activation of potassium channels have also been observed [19]. Nevertheless, controversy exists as not all studies provide a clear and conclusive idea regarding the benefits of PUFAs against cardiovascular diseases [20].

On the other hand, it is well established that sex hormones regulate vascular function by altering the release and cell signaling pathways of endothelial factors that could lead to vascular dysfunction. In this regard, we have demonstrated that the loss of gonadal function modifies the production of vasoconstrictor prostanoids [21–24], superoxide anion [25–27] and NO [28]. Since the number of vascular pathologies matching with decreased levels of sex hormones is increasing [29,30], it would be of interest investigate whether a DHA-supplemented diet could prevent vascular changes induced when an impaired gonadal function exist (i.e.: aging, hypogonadism, and pharmacological treatment of prostate cancer). Therefore, the objective of this work is to analyze how a DHA-supplemented diet affects the lipid profile, the blood pressure, the production of prostanoids and NO, and the redox status of biological samples from control and orchidectomized rats. The vasodilator response and the contribution of NO, prostanoids and hyperpolarizing factors were also analyzed.

## Materials and Methods

### Animals, diets and experimental groups

The protocol was approved by the Animal Ethics Committee of the Universidad Autónoma de Madrid (Ref. CEI-37-829) and procedures were performed according to the European Union directives 63/2010UE and Spanish regulation RD 53/2013.

Male Sprague-Dawley rats (4 months old) were housed in the Animal Facility of the Universidad Autónoma de Madrid (Registration number ES-20079-0000097) under 12 hour light/dark cycles and standard feeding with fodder and water *ad libitum*. After 1 week of adaptation animals were fed a maintenance diet for rodents (Global Diet 2014, Harlan Laboratories Inc. Indianapolis, Indiana, USA) supplemented with fat (5%). The controls-diet groups were supplemented with sunflower oil (5%) and the DHA groups with 4.5% Marinol C-38 (lipid Nutrition) and adjusted to 5% with sunflower oil. Nutrient content and energy distribution of each diet is summarized in Table 1. After 2 weeks on the control- or DHA-diet, animals were divided into two subgroups: control (C) and orchidectomized (ORX) male rats. Male sex hormone deprivation was induced by orchidectomy at 18 weeks of age under anesthesia by isofluorane inhalation. Rats were treated with 0.30 mg/kg meloxicam SC (Metacam 5 mg/ml; Boehringer-Ingelhelm) immediately after surgery and with 50 mg/kg ibuprofen, via oral administration for 4 days. Animals were maintained on experimental diets for six more weeks. At the end of the treatment, rats were sacrificed (6 months old) by CO_2_ inhalation and decapitation. The observation of seminal vesicles atrophy confirmed successful surgery. The aorta was carefully dissected out, cleaned of connective tissue and placed in Krebs-Henseleit solution (KHS) (containing, in mM: NaCl 115, CaCl_2_ 2.5, KCl 4.6, KH_2_PO_4_ 1.2, MgSO_4_ 1.2, NaHCO_3_ 25, glucose 11,1, Na_2_ EDTA 0.03) at 4°C. The investigation conforms to the *Guide for the Care and Use of Laboratory Animals* published by the USA National Institutes of Health (NIH publication No. 85.23 revised 1985). This study was approved by the Ethical Committee of the Universidad Autónoma de Madrid.

**Table 1:** Nutrient and energy content of experimental diets.

	Control Diet	DHA Diet
Carbohydrates (g/100g)	59.84	59.84
Protein (g/100g)	14.39	14.39
Total Fat (g/100g)	9.19	9.19
DHA + EPA		2.01
Energy (kcal/100g)	271.78	271.78

### Blood pressure measurement

Systolic blood pressure was indirectly measured in awake animals by the tail-cuff method [23,24] before and after the treatment using a Letica Digital Pressure Meter LE5000 (Barcelona, Spain).

### Blood biochemical analysis

Triglycerides, total cholesterol, LDL cholesterol, HDL cholesterol, glucose and creatinine in plasma were determined using an automated analyzer (Beckman Coulter-Former Olympus Diagnostics AU 5420, Nyon, Switzerland).

### Release of prostanoids

After a stabilization period in KHS at 37°C for 30 minutes (pH 7.4), aortic rings from each group of rats was followed by 2 wash periods of 10 min using 0.2 mL of KHS. Once fresh KHS was replaced, arteries were exposed to 1 μM noradrenaline (NA) for 2 minutes and then to cumulative ACh concentrations (0.1 nM-10 μM) at 1-minute interval. Then, the medium was collected and stored at -80°C until used. Production of TXA_2_, PGI_2_, PGF_2α_ and PGE_2_ induced by ACh, were monitored by measuring their stable metabolite TXB_2_, 6-keto-PGF_1α_, 13, 14-dihydro-15-keto PGF_2α_ and PGE_2_, respectively, using the respective enzyme immunoassay kit (Cayman Chemical). Results were expressed as pg prostanoid/mg tissue.

### Production of nitric oxide

The fluorescent probe 4,5-diaminofluorescein was used to specifically evaluate NO production, as previously reported [25,26]. Briefly, aortic segments from each group of rats were cryoprotected with 30% w/v sucrose in PBS, frozen and stored at -80°C. After a washing period with PBS, the artery segments were opened to uncover the artery lumen to allow a better penetrance of the probe. Then the segments were immersed in N-(2-hydroxyethyl)piperazine-N0 2-ethane-sulfonic acid (HEPES) buffer (in mM: NaCl 119, HEPES 20, CaCl_2_ 1.2, KCl 4.6, KH_2_PO_4_ 0.4, MgSO_4_ 1, NaHCO_3_ 5, glucose 5.5, Na_2_H_2_PO_4_ 0.15; pH 7.4) containing 4,5-diaminofluorescein (0.5 μM), and incubated in a light-protected, humidified chamber at 37°C for 45 min. Then, the segments were mounted on glad slides and imaged on a confocal microscope. Images were obtained with a LEICA (TCS ST2 DM IRE2) laser scanning confocal microscope (excitation 495 nm, emission 515 nm). Laser and image settings were unchanged for the acquisition of images from the three groups of rats. The photomicrographs show the intensity and location of 4,5-diaminofluorescein, which reflects NO production, so that comparison of these groups could be made. To analyze fluorescence intensity the ImageJ Analysis Software (National Institutes of Health) was used. Although DAF-2 is widely used as detector for NO, it can also react with intermediate products of the oxidation of NO. Therefore, in previous studies, experiments were performed in the presence of the NO synthesis inhibitor L-NAME, in which the fluorescence was abolished [26,31]. The amount of NO released was expressed as arbitrary units.

The basal production of NO was also measured by using a nitrite colorimetric assay kit (Cayman Chemical). Briefly, after a stabilization period in KHS at 37°C for 30 minutes (pH 7.4), aortic rings from each group of rats was followed by 2 wash periods of 10 min using 0.2 mL of KHS. Then, the medium was collected and stored at -80°C until assay performed. The assay was carried out according to the manufacturer’s protocol and the absorbance was measured at 540 nm. Data were expressed as relative levels to control rats fed control-diet (= 1).

### Detection of superoxide anion

Hydroethidine, an oxidative fluorescent probe, was used to evaluate superoxide anion levels *in situ*, as previously described [25,27]. Aortic segments from the four groups were cryoprotected with 30% (w/v) sucrose in PBS, frozen and stored at -80°C. After a washing period with PBS, the artery segments were opened to uncover the artery lumen to allow a better penetrance of the probe. Then the segments were immersed in HEPES buffer containing hydroethidine (5 μM), and incubated in a light-protected, humidified chamber at 37°C for 30 min. Segments were mounted on glad slides and imaged on a confocal microscope. Images were obtained with a LEICA (TCS ST2 DM IRE2) laser scanning confocal microscope (excitation 488 nm, emission 610 nm). Laser and image settings were unchanged for the acquisition of images from the three groups of rats. The photomicrographs show the intensity and location of hydroethidine, which reflects superoxide production, so that comparison of these groups could be made. To analyze fluorescence intensity the ImageJ Analysis Software (National Institutes of Health) was used. In a previous study, we reported that the fluorescence emitted by HE came from superoxide production since the fluorescence diminished in vessels pretreated with tempol, a membrane-permeable of SOD [25]. The amount of superoxide formation was expressed as arbitrary units.

### Detection of hydrogen peroxide

Hydrogen peroxide levels in serum samples and aortic rings were measured by using a fluorescence H_2_O_2_ assay kit (Cayman Chemical). Serum samples from each group of rats were collected upon the day of sacrifice and, frozen at -80°C and used directly to perform the assay. Frozen samples of aortic segments were homogenized at 4°C in RIPA buffer containing 50 mM Tris-HCl (pH 8), 150 mM NaCl, 1% (w/v) deoxycholic acid, 1% (v/v) NP-40, 1% (v/v) SDS, 100 mM NaF, 1 mM Na3VO4, and a protease inhibitor cocktail (Calbiochem) supplemented with freshly prepared 1 mM phenylmethylsulfonyl fluoride. Samples were centrifuged at 16,000 g during 30 min at 4°C and the supernatant was collected and used to the assay, which was carried out according to the manufacturer’s instructions. The fluorescence at 530 and 590 nm excitation and emission wavelengths, respectively, was registered in a 96-microplate reader (Multiskan Ascent, Labsystems). Some assays were performed in the presence of catalase, an H_2_O_2_ scavenger, to ensure the specificity of the method. Supernatants were also used to quantify protein concentration by the bicinchoninic acid assay using the BCA^TM^ Protein Assay Kit (Pierce). Data were expressed as μmol/μg protein for serum samples and nmol/mg protein for aortic tissue.

### Detection of oxygen radical scavenging capacity (ORAC)

The antioxidant activity in serum samples and in aortic vascular wall was analyzed by using the hydrophilic oxygen radical scavenging capacity (ORAC) assay, as previously reported [32]. The sample processing was identical to that explained in the preceding section. The assay was carried out according to the manufacturer’s instructions and the fluorescence read at 485 and 528 nm excitation and emission wavelengths, respectively (Multiskan Ascent, Labsystems). Data were expressed as values relative to control rats fed control-diet.

### Vascular reactivity

The method used for isometric tension recording has been previously described [21,27,33]. In summary, aortic segments were suspended in an organ bath containing 5 mL of KHS at 37°C, continuously bubbled with 95% O_2−_5% CO_2_ mixtures (pH 7.4). Two parallel stainless steel pins were introduced through the lumen of the vascular segment: one fixed to the bath wall and the other connected to a force transducer (Grass FTO3C; Grass Instruments Co., Quincy, MA, USA); this in turn was connected to a model 7D Grass polygraph. The segments were subjected to a tension of 1 g which was re-adjusted every 15 min during a 90 min equilibration period before drug administration. After this, the vessels were exposed to KCl (75 mM) to check the functional integrity. After a washout period the viability of vascular endothelium was tested by the ability of 10 μM ACh to relax precontracted segments with 0.1 μM NA.

Concentration-response curves to ACh (0.1 nM-10 μM) and to the nitric oxide donor sodium nitroprusside (SNP, 0.1 nM-10 μM) were performed in NA (0.1 μM) precontracted aortic rings from of the four groups of rats. To analyze the participation of NO and COX-derivatives on the response induced by ACh, the NO synthase inhibitor L-NAME (0.1 mM) and the nonselective inhibitor of COX-1/2, indomethacin (Indo, 10 μM), were added to the bath 30 minutes before performing the curve. To analyze the participation of hyperpolarizing factors on the responses to SNP and ACh, arteries were precontracted with 30 mM KCl, to block membrane hyperpolarization. To analyze the function of the K_Ca_ and K_ATP_ channels, concentration-response curves to the K_Ca_ and K_ATP_ channel openers NS1619 (0.1 nM-10 μM) and pinacidil (0.1 nM-10 μM) respectively, were also performed in NA (0.1 μM) precontracted aortic rings from each group of rats.

### Reagents

Drugs used were: DAF-2, HE, L-NA hydrochloride, ACh chloride, L-NAME hydrochloride, indomethacin, potassium chloride, SNP, NS1619 and pinacidil (Sigma-Aldrich). Stock solutions (10 mM) of drugs were prepared in distilled water, except for NA which was dissolved in NaCl (0.9%)-ascorbic acid (0.01% w/v) solution, indomethacin in 1.5 mM NaHCO_3_ and NS-1619 in dimethylsulfoxide. These solutions were kept at -20°C and appropriate dilutions were made in KHS on the day of the experiment.

### Statistical analysis

Results are given as mean ± SEM (Standard Error of the Mean). The relaxation induced by ACh or SNP was expressed as a percentage of initial contraction elicited by NA. Statistical analysis was performed by comparing the curve obtained in the presence of the different substances with the control curve by means of two-way analysis of variance (ANOVA). For blood pressure, body weight, biochemical parameters, prostanoids, NO, superoxide, hydrogen peroxide production and ORAC, statistical analysis was done using Student’s *t*-test for unpaired experiments. A *p* value of less than 0.05 was considered significant.

## Results

### Animal weight, systolic blood pressure and biochemical parameters

Before DHA administration, body weight and blood pressure were evaluated in the four groups of animals, showing no statistically significant differences among the groups (Table 2). After six weeks under the experimental diet, no significant changes in blood pressure were found. All animals had increased in body weight to a similar extent (Table 2). As shown in Table 3, orchidectomy induced a significant increase in total cholesterol concentrations and LDL cholesterol, although HDL cholesterol was not statistically modified. Triglyceride concentrations were also increased by the orchidectomy. The DHA-supplemented diet to the orchidectomized rats decreased the total cholesterol, LDL cholesterol and triglyceride levels. However, the control rats fed the DHA-diet decreased only the total cholesterol concentration. No differences were observed in serum concentration of glucose among groups.

**Table 2:** Systolic blood pressure (mmHg) and body weight (g) in control (C) and orchidectomized (ORX) rats fed with a control or DHA-supplemented diet.

	Blood pressure (mm Hg)		Body weight (g)	
**Animal group**	Before diet	After diet	Before diet	After diet
C Control (9)	149.6 ± 3	148.2 ± 3	390.9 ± 10	502.4 ± 20
C DHA (7)	145.4 ± 4	142.4 ± 6	394.9 ± 7	484.9 ± 12
ORX Control (8)	150.2 ± 2	146.4 ± 5	385.4 ± 8	477.5 ± 18
ORX DHA (11)	146.6 ± 3	143.5 ± 1	411.6 ± 10	506.5 ± 9

Values are means ± SEMs. The number of animals used is indicated in parenthesis.

**Table 3:** Serum lipid profile and glucose concentration in control (C) and orchidectomized (ORX) rats fed with a control or DHA-supplemented diet.

Serum biomarkers (mg/dL)	C Control	C DHA	ORX Control	ORX DHA
Cholesterol	121.4 ± 5.55	89.0 ± 14.57^a^	153.6 ± 6.49^a^	91.25 ± 6.42^b^
HDL Cholesterol	68.25 ± 4.29	59.28 ± 6.63	78 ± 4.68	70.2 ± 5.85
LDL Cholesterol	33.15 ± 3.12	26.13 ± 5.07	49.92 ± 3.9^a^	24.18 ± 2.73^b^
Triglycerides	99.20 ± 5.74	80.33 ± 18.89	141.8 ± 9.63^a^	83.60 ± 6.8^b^
Glucose	121.2 ± 12.85	150.3 ± 8.5	127.0 ± 8.4	113.0 ± 20.81

Values are mean ± SEMs. Number of animals per group, n = 5.

^a^ Indicates difference with C Control group values.

^b^ Indicates differences with the ORX group values.

### Release of prostanoids

**Fig 1:**
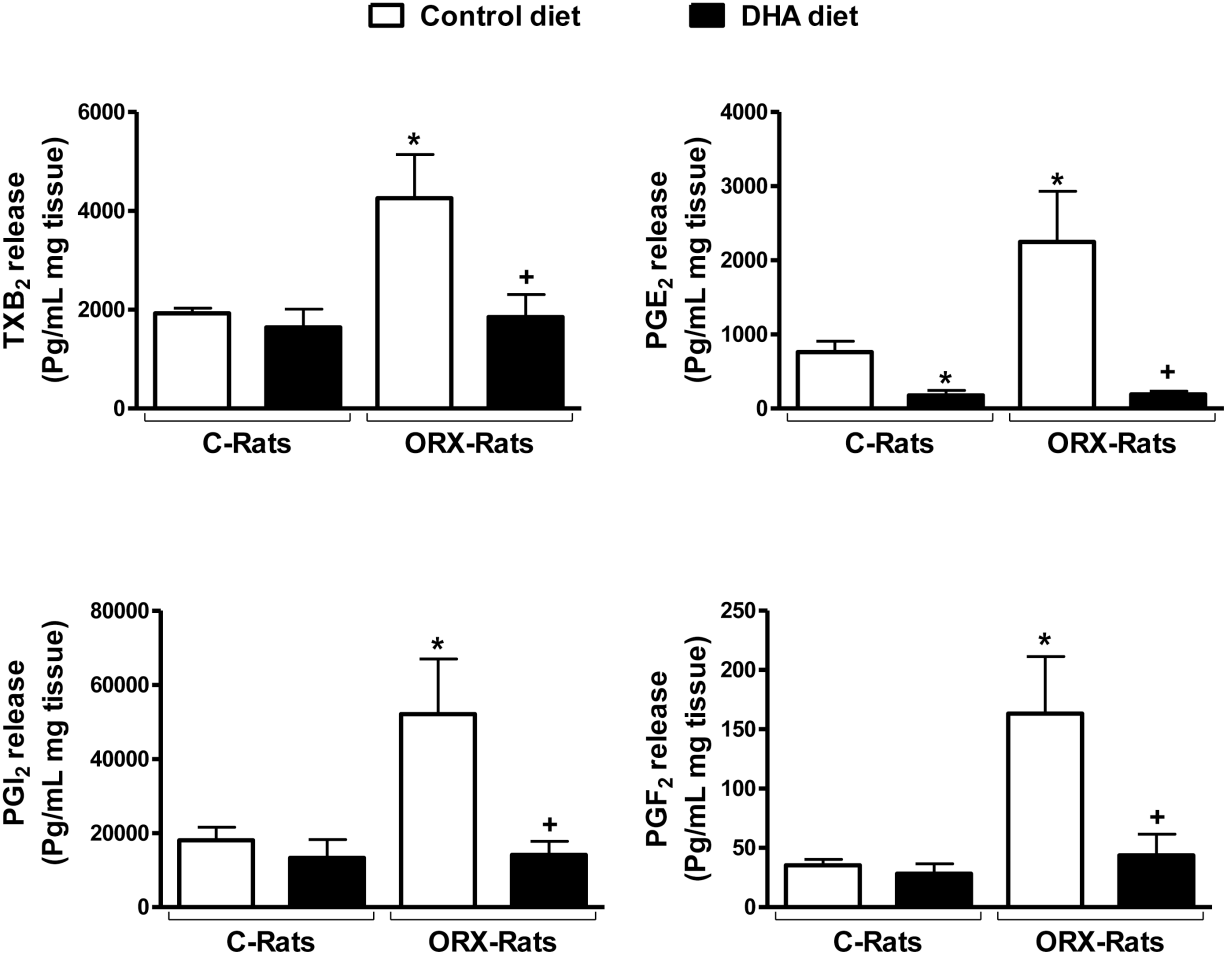
Effect of orchidectomy and a DHA-supplemented diet on basal release of prostanoids from rat aorta. Release of thromboxane B_2_ (TXB_2_), prostaglandin E_2_ (PGE_2_), PGI_2_ and PGF_2α_ in aortas from control (C) and orchidectomized (ORX) rats fed with a control or with a DHA-supplemented diet. Values are means ± SEMs. Number of animals, n = 5. *P < 0.01 compared with C rats fed Control diet; *+P* < 0.01 compared with ORX rats fed DHA-diet.

Orchidectomy increased the ACh-stimulated aortic production of TXA_2_, PGI_2_, PGF_2α_ and PGE_2_ and PGE_2_ (Fig 1). These values were restored to similar levels found in aorta from control animals after dietary DHA supplementation. In contrast, the DHA-diet in control rats decreased PGE_2_ release and did not significantly modify the release of TXA_2_, PGI_2_, PGF_2α_ (Fig 1).

### Production of nitric oxide

**Fig 2:**
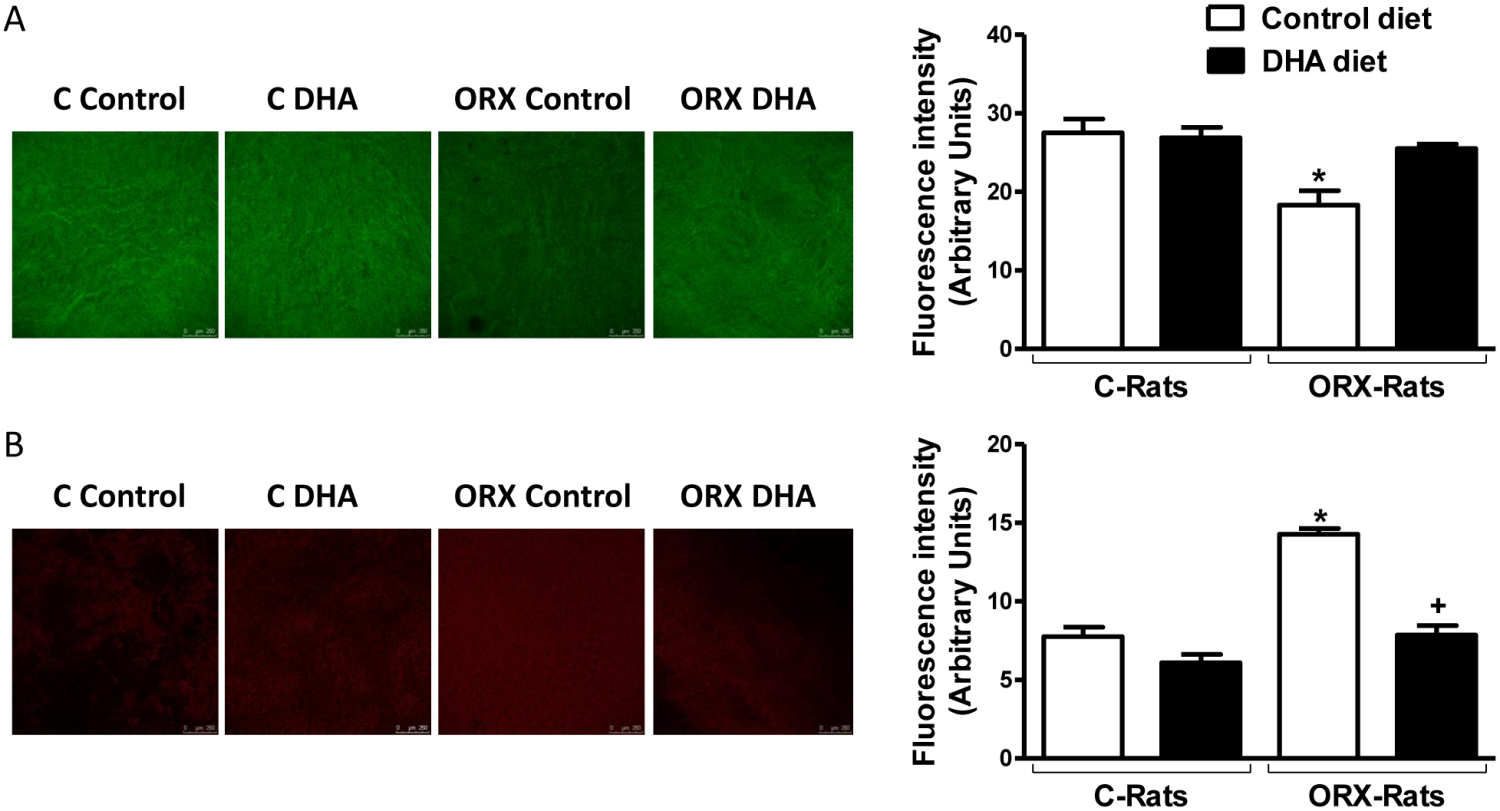
Effect of orchidectomy and the DHA-supplemented diet on the production of endothelial NO and superoxide anion from rat aorta. Confocal micrographs showing in situ detection of NO (A) or superoxide anion (B) in aortic segments from control (C) and orchidectomized (ORX) rats fed with a control diet or with a DHA-supplemented diet (DHA). The sections shown are typical preparations from five rats. Quantitative analysis of fluorescence is also shown. Values are means ± SEMs. Number of animals, n = 5. *P < 0.01 compared with control animals; *+P* < 0.01 compared with ORX rats control diet.

After incubation with 4,5-diaminofluorescein, the fluorescence emitted was decreased in arteries from orchidectomized rats respect to arteries from control rats, and it was recovered after DHA-supplemented diet to orchidectomized rats; however, the DHA-diet did not modify the fluorescence emitted in arteries from control rats (Fig 2A). Relative basal measurement of nitrite levels shown similar results (arteries from control rats-control diet: 1; orchidectomized rats-control diet: 0.14 ± 0.06, *p* < 0.05 *vs* control diet; control rats-DHA diet: 0.98 ± 0.3, *p* > 0.05 vs control diet; orchidectomized rats-DHA diet: 0.77 ± 0.15, *p* < 0.005 vs control diet; n = 5).

### Detection of superoxide anion

After incubation with hydroethidine, the arteries from orchidectomized rats showed a markedly higher level of fluorescence than the arteries from control rats. This increase was reduced in the vessels from orchidectomized rats fed with the DHA-supplemented diet. In contrast, the DHA-diet did not affect the production of superoxide anion in control animals (Fig 2B).

### Detection of hydrogen peroxide

**Fig 3:**
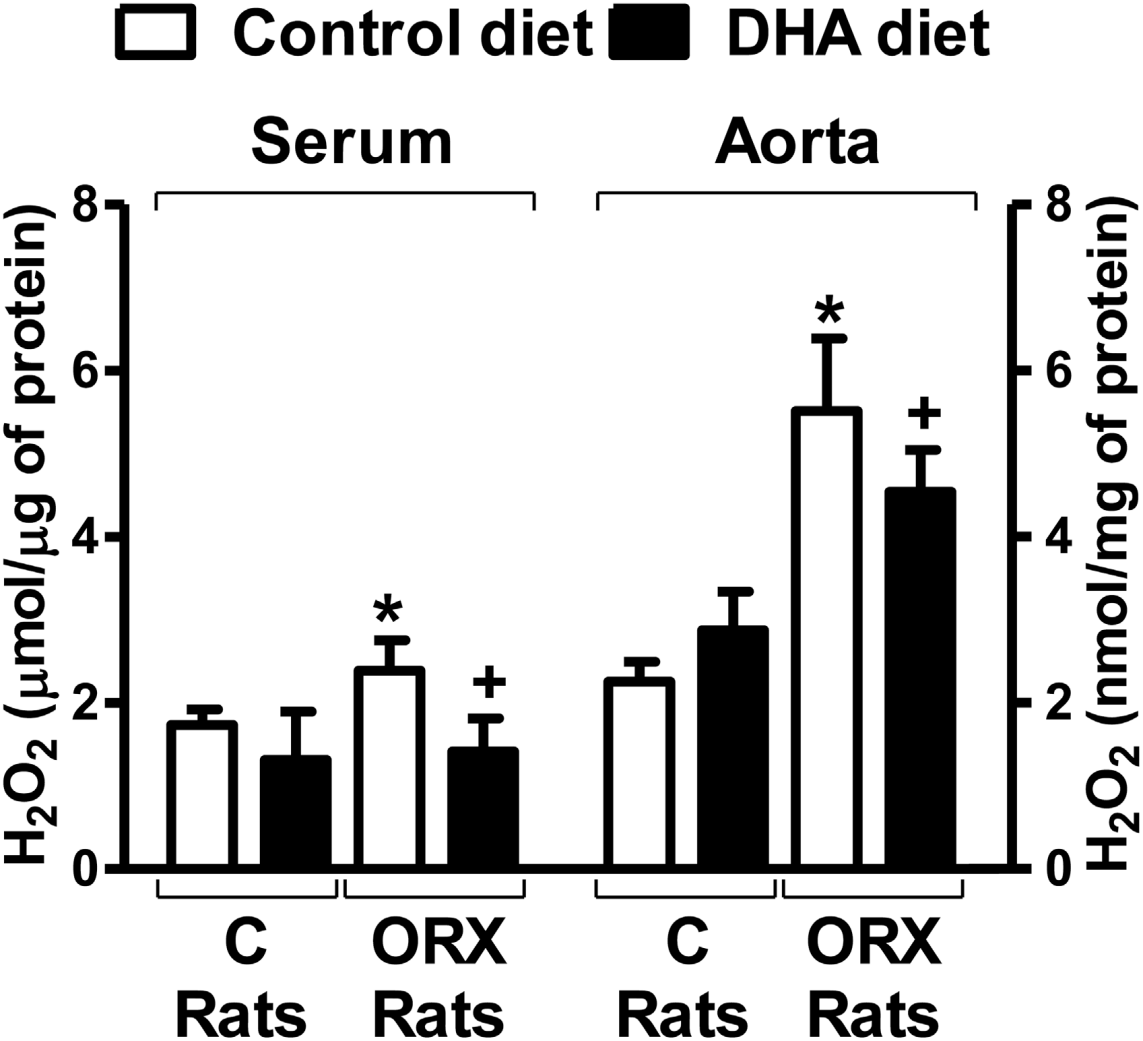
Effect of orchidectomy and the DHA-supplemented diet in the H_2_O_2_ content in rat serum and aorta. Relative levels of H_2_O_2_ in serum and aortic rings from control (C) and orchidectomized (ORX) rats fed with a control or with a DHA-supplemented diet. Values are means ± SEMs. Number of animals, n = 4. *P < 0.01 compared with control animals; *+P* < 0.01 compared with ORX rats control diet.

The levels of hydrogen peroxide, in serum samples and aortic wall, were increased in orchidectomized rats, which were returned to control level when orchidectomized rats fed with the DHA-supplemented diet. The DHA-diet to control animals did not modify hydrogen peroxide levels (Fig 3).

### Antioxidant activity

**Fig 4:**
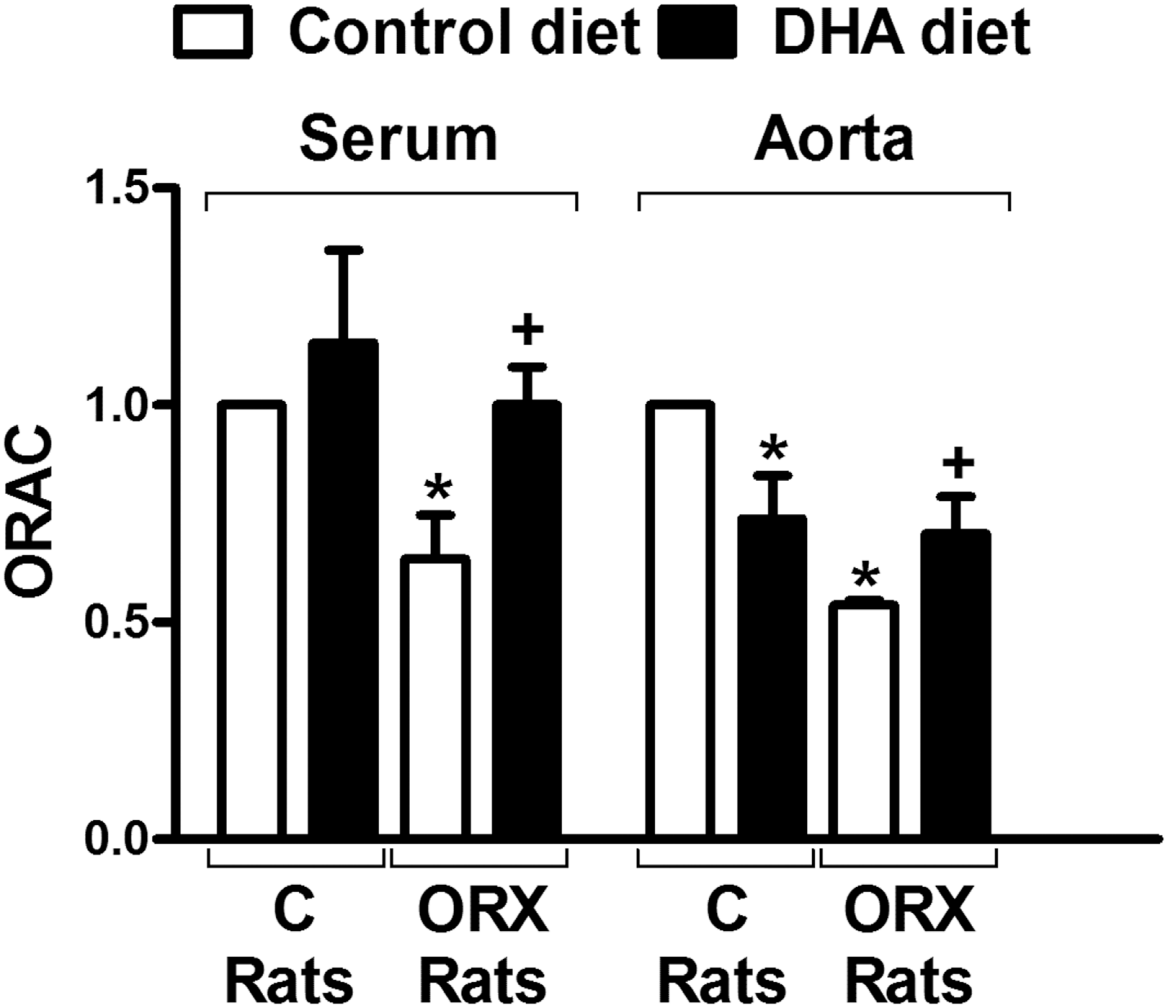
Effect of orchidectomy and the DHA-supplemented diet in the antioxidant activity from rat serum and aorta Relative levels of H_2_O_2_ in serum and aortic rings from control (C) and orchidectomized (ORX) rats fed with a control or with a DHA-supplemented diet. Values are means ± SEMs. Number of animals, n = 6. *P < 0.01 compared with control animals; *+P* < 0.01 compared with ORX rats control diet.

The antioxidant activity in both serum and aortic wall was decreased by the orchidectomy. DHA-diet to orchidectomized rats restored the activity up to levels similar shown in the control rats. However, the DHA-diet failed to modify the antioxidant activity in the serum from control animals, and surprisingly decreased the activity level in the aortic vascular wall (Fig 4).

### Vascular reactivity

**Fig 5:**
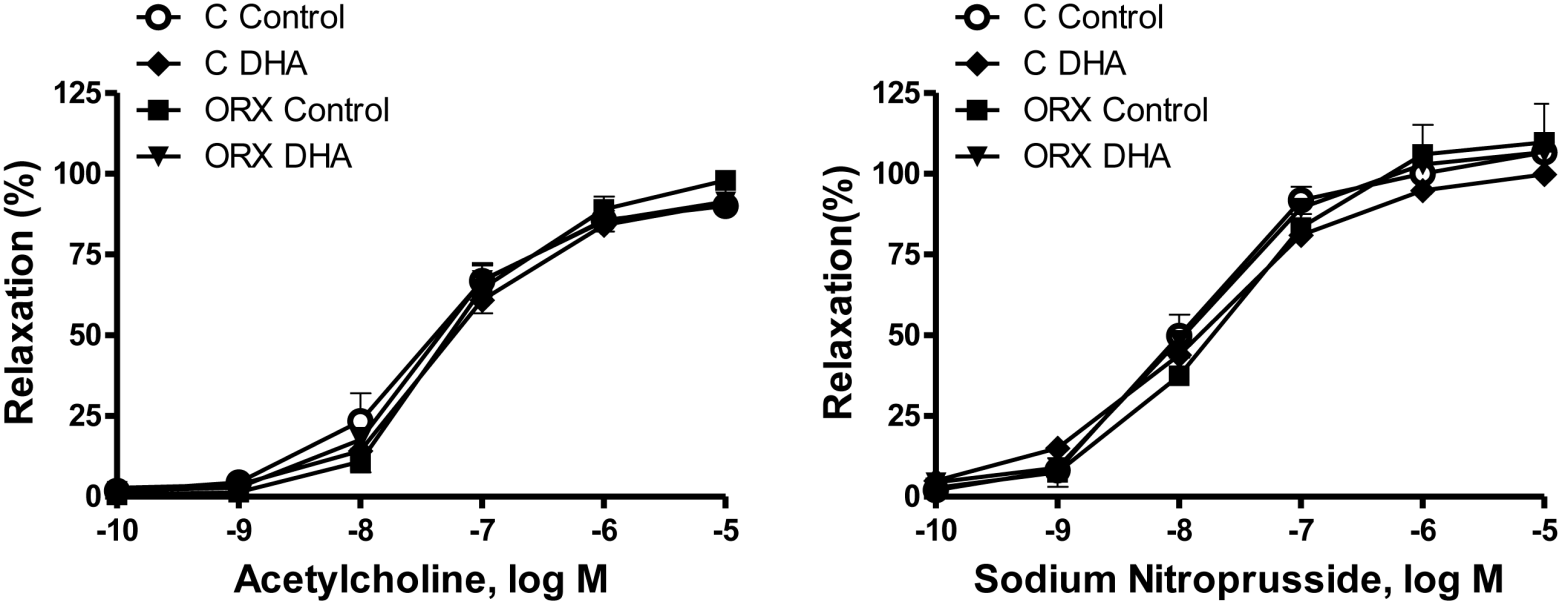
Effect of orchidectomy and a DHA-supplemented diet on the concentration-response curves to acetylcholine and sodium nitroprusside in rat aorta. Concentration-response curves to acetylcholine and sodium nitroprusside in aortic segments from control (C) and orchidectomized (ORX) rats fed with a control or a DHA-supplemented diet. Results (means ± SEMs) are expressed as percentage of inhibition of the contraction induced by 0.1 μM noradrenaline. Number of animals, n = 5–8.

The vasodilator response induced by ACh (0.1 nM-10 μM) in aortic segments precontracted with NA (0.1μM) was similar in the four groups of rats (Fig 5). The vasodilator response induced by the NO donor SNP was not modified in any group (Fig 5), indicating that the sensitivity of NO on smooth muscle was intact.

**Fig 6:**
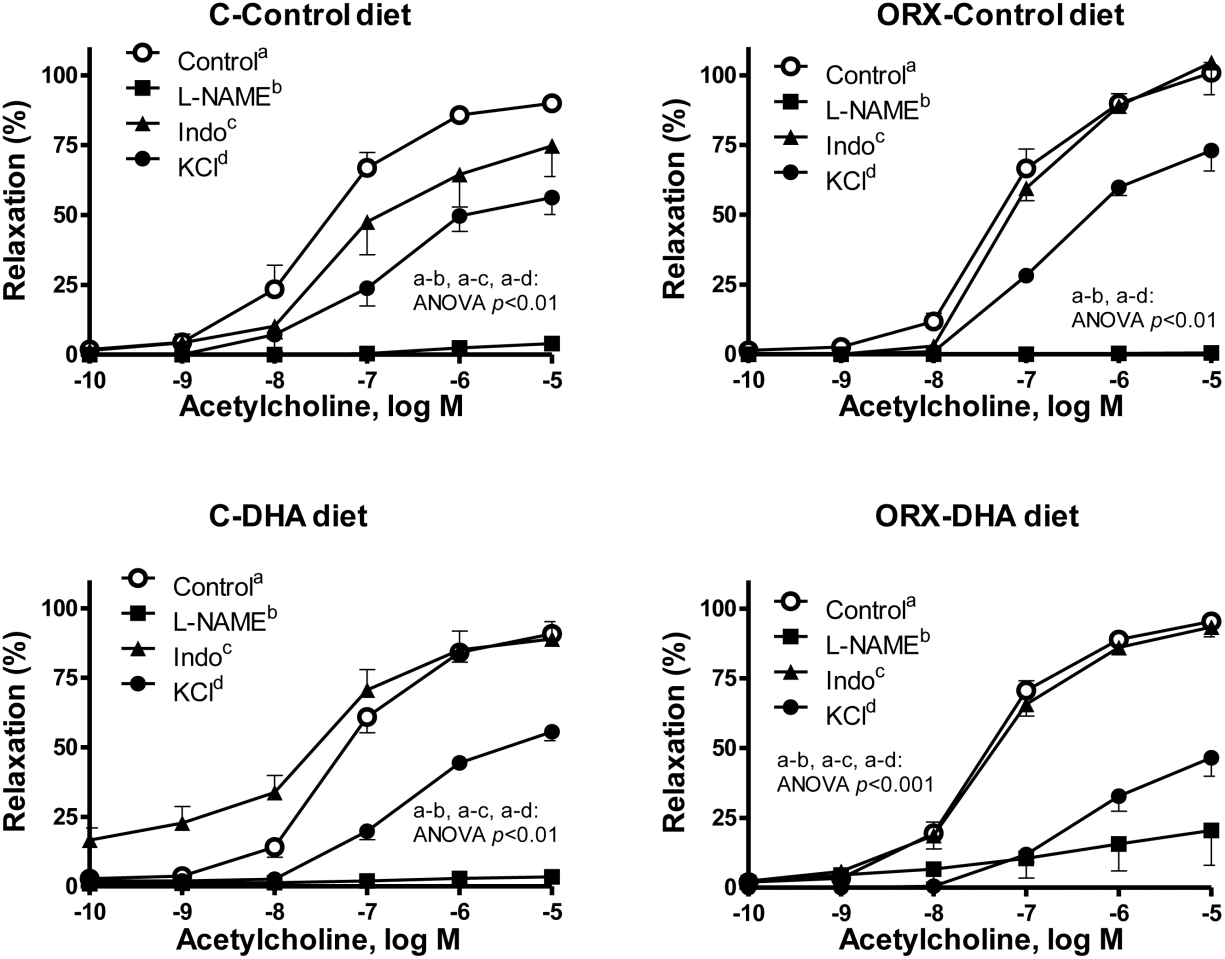
Orchidectomy and DHA-supplemented diet modulate the participation of different factors in the Ach-induced responses. Effect of L-NAME (0.1 mM) or indomethacin (Indo, 10 μM) on the concentration response curves to acetylcholine in the NA-precontracted aortic segment from control (C) and orchidectomized (ORX) rats fed with a control diet or with a DHA-supplemented diet (DHA diet). The effect of precontracting vessels with KCl (30 mM) is also represented. Results (means ± SEMs) are expressed as percentage of inhibition of the contraction induced by 0.1 μM NA or 30 mM KCl. Number of animals, n = 5–8.

To investigate the contribution of NO or prostanoids on the vasodilator response induced by ACh, the effect of the inhibitors of NO or prostanoids synthesis, L-NAME or indomethacin, was examined. In the presence of L-NAME the ACh-induced relaxation was inhibited in aortic segments from control or orchidectomized rats; the control-diet did not alter the inhibitory effect of L-NAME, while in segments from orchidectomized rats supplemented with DHA a small relaxation occurred (Fig 6). Incubation with indomethacin decreased the ACh-induced relaxation response in arteries from control rats fed control diet, while it did increase the ACh-induced response when the animals were fed DHA-diet. In aortas from orchidectomized rats fed control- or DHA-diet, indomethacin did not modify the Ach-induced response (Fig 6).

**Fig 7:**
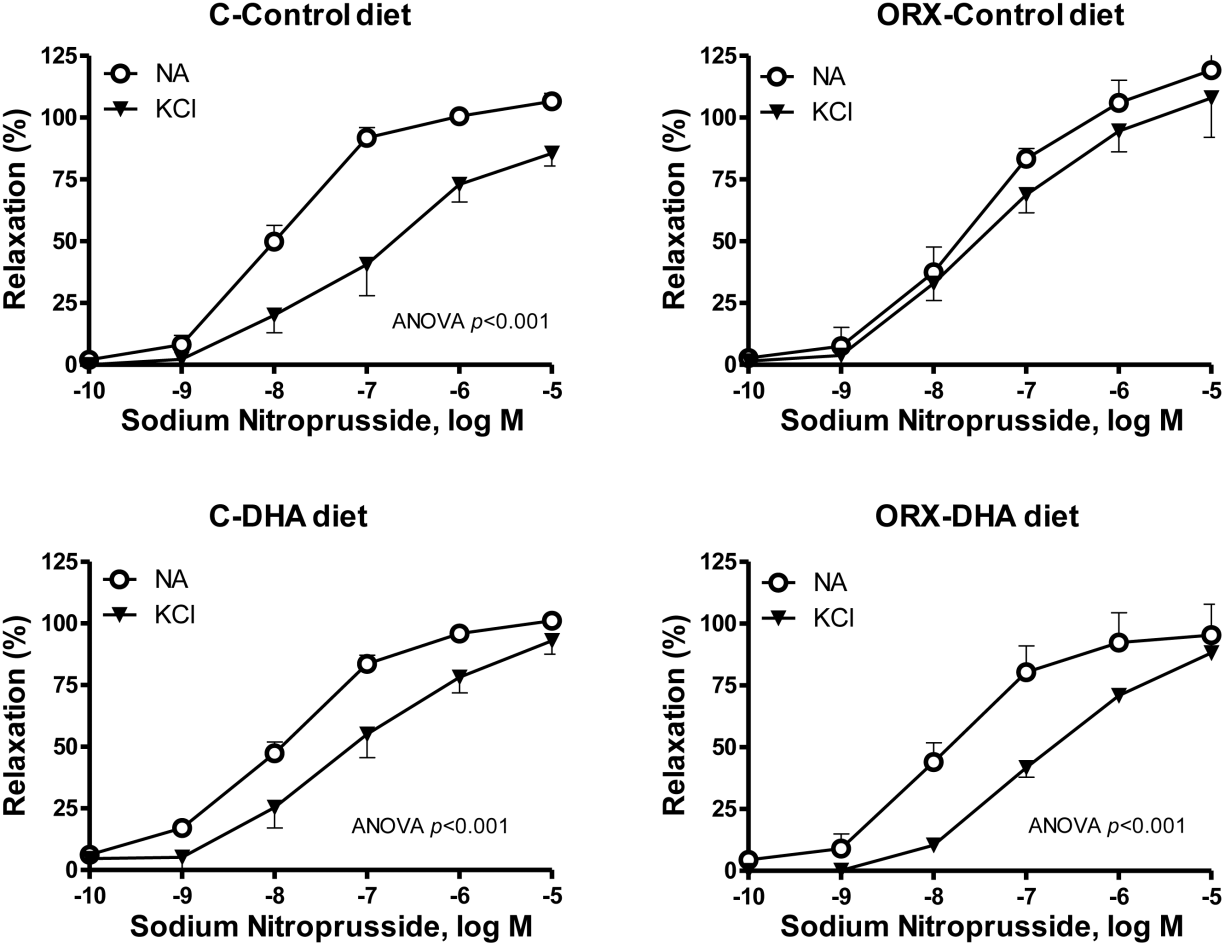
Orchidectomy and DHA-supplemented diet modulate the involvement of hyperpolarizing mechanisms in the sodium nitroprusside-induced response. Concentration-response curves to sodium nitroprusside in NA- or KCl-precontracted aortic segments from control (C) and orchidectomized (ORX) rats fed with a control diet or with a DHA-supplemented diet (DHA diet). Results (means ± SEMs) are expressed as percentage of inhibition of the contraction induced by 0.1 μM NA or 30 mM KCl. Number of animals, n = 4–7.

The possible participation of hyperpolarizing mechanisms in the ACh-induced response was also analyzed by precontracting vessels with KCl (30 mM) that blocks the membrane hyperpolarization. Under this condition the ACh-induced response was decreased in arteries from the four groups of animals (Fig 6), but in a greater extent in aortas from orchidectomized animals fed with a DHA supplemented diet. Since NO can also hyperpolarize the membrane of smooth muscle cell, the participation of hyperpolarizing mechanisms on the relaxation induced by SNP was also analyzed. In KCl-precontracted arteries the relaxation induced by SNP was reduced in the four groups of animals (Fig 7). The SNP-induced relaxation was decreased in a greater extent in arteries from control rats than in those from orchidectomized rats. The DHA-diet did not significantly modify the response in arteries from control rats, while it increased the blockage in arteries from orchidectomized (Fig 7).

**Fig 8:**
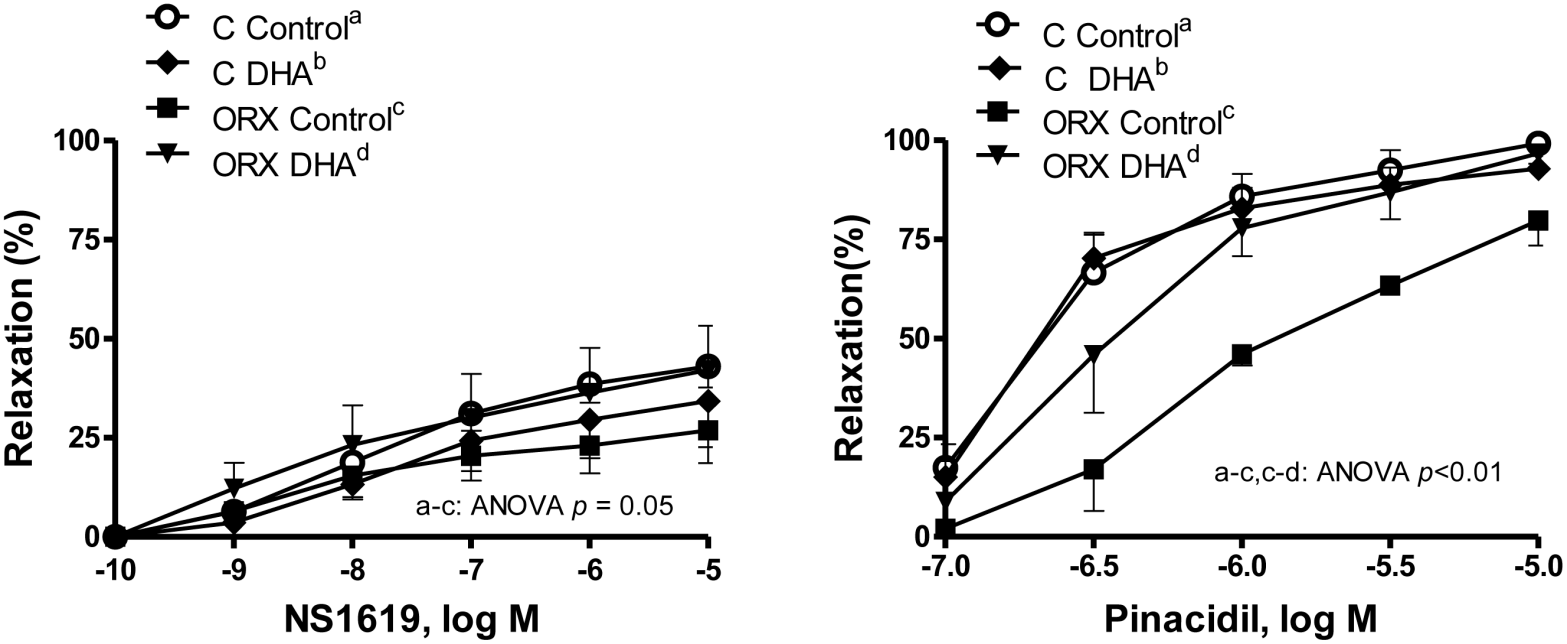
Effect of orchidectomy and the DHA-supplemented diet on the function of K_Ca_ and K_ATP_ channels. Concentration-response curves to the K_Ca_ and K_ATP_ channels openers NS1619 and pinacidi in NA-precontracted aortic segments from control (C) and orchidectomized (ORX) rats fed with a control diet or with a DHA-supplemented diet (DHA). Results (means ± SEMs) are expressed as percentage of inhibition of the contraction induced by 0.1 μM NA. Number of animals, n = 4–6.

Since the activation of the K_Ca_ and K_ATP_ channels causes vasodilation by hyperpolarizing cell membrane, the effects of orchidectomy and DHA supplementation on the function of the K_Ca_ and K_ATP_ channels were analyzed. Concentration-response curves to the K_Ca_ and K_ATP_ channels openers NS1619 (0.1 nM-10 μM) and pinacidil (0.1 μM-10 μM), respectively, were analyzed in NA (0.1 μM) precontracted aortic rings from the four groups of rats (Fig 8). Orchidectomy partially decreased the vasodilator response induced by NS1619, which was recovered in the orchidectomized DHA group. Orchidectomy decreased the pinacidil-induced response, which was also recovered after DHA-supplemented diet. However, the DHA-diet failed to modify the NS1619- and pinacidil-induced responses in arteries from control rats (Fig 8).

## Discussion

The present work provides information on the different impact of a DHA-supplemented diet to healthy or orchidectomized rats, analyzing the possible modifications in the lipid profile, in the release of different vascular factors and in the redox status that control vascular function. The interest of this particular experimental model is because the number of vascular pathologies matching with decreased levels of sex hormones (i.e.: aging, hypogonadism, and pharmacological treatment of prostate cancer) is increasing [29,30]. We have previously demonstrated that the release of prostanoids was increased 5 months post-orchidectomy in aorta and mesenteric arteries [21–23,25], most likely as a consequence of increased oxidative stress observed in these experimental conditions. In the present study we show that in aorta from 6 weeks post-orchidectomized rats the ACh-induced release of PGI_2_, PGF_2α_ and PGE_2_ were already increased, as observed for TXA_2_ release in a previous study [28]. These results showed that the loss of gonadal function favored the onset of a pro-inflammatory environment. Dietary supplementation with DHA prevented the increase in the prostanoids release induced by orchidectomy, which agrees with the described anti-inflammatory properties of PUFAs [16,17,34]. However, in control animals, this property was shown for PGE_2_, that was decreased, which is in line with most of the studies describing a reduction after PUFAs-diet in healthy subjects [35,36]. The DHA-diet did not modify the production of TXA_2_, PGI_2_ and PGF_2α_ in control rats, as previously reported for TXA_2_ production [37].

Regarding the production of NO, quantified by measuring nitrite production and the fluorescence emitted by DAF-2, the current study shows that it was decreased in aortas from 6 weeks post-orchidectomized rats, according to previously published [28], while in aortas from orchidectomized rats fed a DHA-supplemented diet the NO production was restored. These results are in line with other studies describing increase in NO release by n-3 PUFA [38–40]. However, the DHA-diet did not significantly modify the release of NO in arteries from control rats, as reported by Omura and coworkers [41] who observed that although the EPA increased NO release, DHA had no effect. This result indicates that the effect on NO release may depend on the specific PUFA tested. But what is more important is that the effect of a specific PUFA, DHA in our study, on the prostanoids and NO release seems to depend on the baseline conditions of the experimental treated animals.

Oxidative stress is one of the major detrimental events in the induction of vascular dysfunction. Since we already reported that 5 months post-orchidectomy increased the production of superoxide anion [25,26,31], we now analyzed the effect of the DHA-supplemented diet on the ROS production. The results showed that 6 weeks post-orchidectomy already increased the production of superoxide in the aortic wall, while in arteries from orchidectomized rats fed the DHA-supplemented diet the production of superoxide was diminished to a level similar of that found in arteries from control rats. However, the aorta from control rats fed DHA-diet did not modify the production of superoxide. Thus, superoxide production has been decreased by n-3 PUFAs in platelets from diabetic hypertensive patients [42]. DHA protects endothelial cells culture against oxidative stress through Nrf2 activation [43] and down-regulating Nox4 [44]. These results suggest that DHA interfere with the oxidative stress in pathophysiological situations in which there is a ROS overproduction. It is important to mention that although in previous studies we reported that the fluorescence emitted by hydroethidine came from superoxide-derived products [25,27], the specificity of this probe has been questioned because it could generate artefacts [45–47]. Therefore, it would be desirable to use other methods in future studies. Nevertheless, since superoxide can be metabolized to hydrogen peroxide, which was increased in mesenteric arteries from orchidectomized rats [25], and that exerts important vascular effects [25, 27] we also analyzed the presence of this compound in serum and aortic wall. The results shown that orchidectomy increased the hydrogen peroxide content in both serum and aortic samples, which were restored by the DHA-diet. These results are in agreement with the modification in the superoxide anion production commented above. Here again, control rats fed DHA-diet did not modify the hydrogen peroxide content respect to that observed with control diet. In view of these results, the oxygen radical scavenging capacity in serum and aortic wall was also studied. As expected, orchidectomy decreased the antioxidant activity, which was recovered in the orchidectomized rats fed DHA-diet. DHA-diet failed to modify the antioxidant activity in samples from control animals. The overall results on the redox status demonstrates the antioxidant ability of DHA [48,49] and provides evidence for a positive effect of DHA-supplemented diet to prevent vascular dysfunction caused by an imbalance between the formation and elimination of ROS.

In addition to changes in the production of NO, prostanoids and ROS induced by orchidectomy, the lipid profile is also under control of the androgens levels, and importantly affects the vascular function. In this regard, the orchidectomized group fed the control-diet showed an increase in total cholesterol, LDL-cholesterol and triglycerides, according to the effect of androgen deprivation on lipid profile [50]. However, the DHA-diet normalized the lipid levels in orchidectomized rats while in control animals diminished only the total cholesterol level, without modifying the rest of parameters. These results are in line with previous human [51] and animal [52] studies showing that PUFAs intake improves the altered lipid profile.

Importantly, despite the changes in the production of NO, prostanoids, ROS and in the lipid profile in the ORX and ORX-DHA group, the systolic blood pressure was not modified in any group. The lack of effect of a DHA-supplemented diet on blood pressure is in agreement with other studies where no effects were noted in the short term [53]. However, a decrease in blood pressure has also been reported in both spontaneously hypertensive [54,55] and aged [53] rats. Additionally, our results also suggest that the alterations in vascular function described below are independent of hemodynamic changes.

Regarding endothelium-dependent relaxation, it was shown that 6-weeks post-orchidectomy did not modify the ACh-induced relaxation. The DHA-supplemented diet to control or orchidectomized rats did not alter the ACh-induced relaxation, which is in agreement with other studies in aged [53], hypertensive [55] or diabetic [56] rats. However, other studies have revealed that the intake of n-3 PUFAs improves the NO-mediated endothelium-dependent vasodilation in patients with coronary artery disease [57]. These results suggest that the influence of DHA on the endothelium dependent vasorelaxation is controversial and appears to mainly depend on the experimental conditions and the animal species.

It is noteworthy that while the release of prostanoids and NO are modified by the orchidectomy and restored by the DHA-diet, the endothelial-dependent vasodilator responses induced by ACh remain unchanged in vessels from the four groups of rats. However, the contribution of different factors such as NO, prostanoids or hyperpolarizing mechanisms cannot be ruled out. We observed that NOS inhibition with L-NAME blocked the ACh-induced response in aortas from control rats with control- or DHA-diet, indicating that the vasorelaxant response to ACh is mainly due to the NO, as described for large elastic arteries [58]. In aortas from orchidectomized rats, the ACh-induced relaxation was also abolished by L-NAME, which coincides with the diminished production of NO reported in aorta from these animals. However, in aorta from orchidectomized rats fed with DHA, L-NAME did not stop the ACh-induced response, as a very small relaxation can be observed. Considering that the sensitivity of the smooth muscle to NO was not modified, these results indicate that although NO is the most important factor in the response to ACh, other mechanisms may be contributing to the relaxation in this group of vessels. Therefore, the participation of prostanoids in the ACh-induced response were analyzed. The results showed that the inhibition of COX-1 and COX-2 with indomethacin decreased the vasodilator action of Ach in control rats, indicating the involvement of vasodilator net effect of prostanoids in this response. However, in aorta from orchidectomized rats the ACh-induced response was not modified suggesting that the net vasodilator effect has been lost. This is supported by the increased production of vasoconstrictor prostanoids, which was discussed above. Furthermore, when prostanoid synthesis is inhibited the formation of NO is modified [22], indicating that NO may also be involved.

Despite the decreased production of prostanoids, in arteries from orchidectomized rats fed the DHA-diet, indomethacin did not modify the ACh-induced response, which could be attributed to that DHA could reduce the sensitivity of the smooth muscle to prostanoids [59,60]. More surprising was the effect of DHA-diet in control rats, in which an increase in the Ach response in presence of indomethacin was observed, indicating the prevalence of a net vasoconstrictor effect of prostanoids. This result may be related to the decrease in PGE_2_, and indicates that intake of DHA supplements not would have a beneficial effect on the vasodilator response in healthy subjects.

Since the endothelium is able to synthesize EDHF and/or substances that hyperpolarize the cell membrane [8–10], the participation of hyperpolarizing mechanisms in the ACh-induced response was analyzed. Our results show that after blocking hyperpolarization [61], the response to ACh was decreased in all groups of animals, but to a greater extent in aortas from orchidectomized rats with DHA-supplemented diet. This suggests a participation of hyperpolarizing mechanisms in the relaxation induced by ACh, in particular when orchidectomized animals are fed a DHA-supplemented diet. These results are in agreement with the hyperpolarizing mechanisms induced by DHA [17]. In view of these results, the hyperpolarizing effect of NO was also investigated. These results indicate that NO partially hyperpolarizes the cell membrane and that orchidectomy decreases the degree of SNP-induced hyperpolarization, which was restored by the DHA-diet. It is well known that K_Ca_ and K_ATP_ channels have an important role on vascular tone regulation [5,7] and that their function may be modulated by sex hormones [62–64] and PUFAs [17,65,66]. Taking into account that orchidectomy decreases the participation of hyperpolarizing mechanisms induced by ACh or SNP, the vasodilator response to the K_Ca_ and K_ATP_ channels openers NS1619 and pinacidil, respectively, were analyzed. We observed that orchidectomy decreased the NS1619- and pinacidil-induced responses, which is in line with the hyperpolarizing effects caused by the acute administration of testosterone [62–64]. In arteries from orchidectomized DHA-supplemented rats, the vasodilator response elicited by the potassium channels openers was restored up to similar levels to those found in arteries from control rats. Therefore, the effect of DHA supplementation is in agreement with other studies where increased activation of K_Ca_ [19,65,66] and K_ATP_ [67,68] channels have been reported. However, the DHA-diet did not increase the activation of K channels in control to rats, pointing out that the choice of experimental models is crucial to determine the actions of a DHA-supplemented diet.

The results showed in the present study are of pathophysiological relevance since they provided mechanisms involved in the regulation of elastic artery function. The maintenance for long periods of an increased oxidative stress and chronic inflammation increase the proteolytic activity of the extracellular matrix, which could affect the integrity of aortic wall and ultimately lead to vascular damage as observed in aortic aneurysms [69].

In summary, the results obtained in aortas from male rats showed that orchidectomy induced the following effects: i) increased the production of prostanoids and ROS; ii) decreased NO production and the antioxidant capacity; iii) negatively affected the lipid profile; and iv) decreased the participation of hyperpolarizing mechanisms in the vasodilator responses, in which K_Ca_ and K_ATP_ channels are involved. The DHA-supplemented diet of the orchidectomized rats exerted anti-inflammatory and antioxidant effects by decreasing the release of prostanoids and ROS, while increasing NO production and the antioxidant capacity, and it also improved the lipid profile. Additionally, it increased the participation of hyperpolarizing mechanisms by activating K_Ca_ and K_ATP_ channels.

Since the modifications induced by the DHA-supplemented diet were observed only in the orchidectomized but not in the healthy group, the overall results show that DHA exerts cardioprotective effects in physiopathological situations in which vascular dysfunction exists. To further explore the mechanisms of action of PUFAs in health and disease to explain these differences more studies will be required.
